# Periaqueductal gray matter echogenicity as a marker of migraine chronification: a case control study

**DOI:** 10.1186/s10194-023-01576-3

**Published:** 2023-04-17

**Authors:** Dolores Vilas, Sara Rubio, Mireia Gea, Jose Rios, Lourdes Ispierto, María Hernández-Pérez, Martí Paré, Mònica Millán, Laura Dorado

**Affiliations:** 1 Department of Neurosciences, Neurology Service, University Hospital Germans Trias I Pujol, Carretera Canyet s/n, 08916 Badalona Barcelona, Spain; 2 Institut de Recerca Germans Trias I Pujol (IGTP), Univesitat Autònoma de Barcelona, Badalona, Barcelona, Spain; 3 Department of Clinical Farmacology, Hospital Clinic and Medical Statistics Core Facility, Institut d’Investigacions Biomèdiques August Pi I Sunyer (IDIBAPS), Barcelona, Spain; 4 Biostatistics Unit, School of Medicine, Universitat Autònoma de Barcelona, Barcelona, Spain

**Keywords:** Migraine, Chronic migraine, Periaqueductal gray matter, Echogenicity, Transcranial sonography

## Abstract

**Background:**

Migraine is one of the most prevalent and disabling medical diseases in the world. The periaqueductal gray matter and the red nucleus play an important role in its pathogenesis. Our aim was to evaluate the echogenicity of the periaqueductal gray matter and the red nucleus in patients with migraine, by means of transcranial ultrasound.

**Methods:**

In this cross-sectional study, a group of patients with migraine (according to the International Classification of Headache Disorders) and a group of control subjects with comparable age-and-sex distribution were prospectively included. We evaluated the area and echogenicity of the periaqueductal gray matter and the red nucleus by means of transcranial ultrasound, both bedside and posteriorly analyzed with the medical image viewer Horos.

**Results:**

We included 115 subjects: 65 patients with migraine (39 of them with chronic migraine and 26 with episodic migraine), and 50 controls. Median disease duration in patients with chronic migraine was 29 (IQR: 19; 40) years, with a median of 18 (IQR: 14; 27) days of migraine per month. The area of the periaqueductal gray matter was larger in patients with chronic migraine compared to episodic migraine and controls (0.15[95%CI 0.12;0.22]cm^2^; 0.11[95%CI 0.10;0.14]cm^2^ and 0.12[95%CI 0.09;0.15]cm^2^, respectively; *p* = 0.043). Chronic migraine patients showed an intensity of the periaqueductal gray matter echogenicity lower than controls (90.57[95%CI 70.87;117.26] vs 109.56[95%CI 83.30;122.64]; *p* = 0.035). The coefficient of variation of periaqueductal gray matter echogenicity was the highest in chronic migraine patients (*p* = 0.009). No differences were observed regarding the area or intensity of red nucleus echogenicity among groups.

**Conclusion:**

Patients with chronic migraine showed a larger area of echogenicity of periaqueductal gray matter, a lower intensity of its echogenicity and a higher heterogenicity within this brainstem structure compared to patients with episodic migraine and controls. The echogenicity of the periaqueductal gray matter should be further investigated as a biomarker of migraine chronification.

**Supplementary Information:**

The online version contains supplementary material available at 10.1186/s10194-023-01576-3.

## Background

Migraine is one of the most prevalent and disabling medical diseases in the world. The World Health Organization (WHO) classifies migraine as the leading cause of disability between the ages of 15 and 49, and the second leading cause of disability for activities of daily living globally [1]. According to the international classification of headaches (ICHD-3) [2] migraine is chronic (CM) if patients have headache 15 or more days per month, of which at least eight meet criteria for migraine, and for minimum during 3 months [2]. Approximately, 3% of patients with episodic migraine (EM) develop CM each year [3], being patients with CM those with a greater disability [4].

Although the pathophysiology of migraine is not yet fully understood, the trigeminovascular system, the hypothalamus, the brainstem nuclei and the cortex are involved in the generation of migraine headache [5]. Among the brainstem nuclei, the periaqueductal gray matter (PAG) and the red nucleus (RN) play an important role in the pathogenesis of the migraine. The PAG controls nociceptive responses [6], allows the inhibition of the painful stimulus and connects with encephalic structures, ascending medullary fibers and pons' structures as the raphe nucleus magnus [7]. Magnetic resonance imaging studies have demonstrated the presence of iron deposits in the PAG and RN in patients with migraine [8, 9]. These iron depositions, and its size have been correlated with the frequency and intensity of migraine attacks, and the time course of the disorder, suggesting a causal relationship between recurrent attacks and iron accumulation [8, 9].

A precise diagnosis of migraine, especially in CM, may be potentially difficult, since patients might suffer a nonspecific pain that can lead to errors in diagnosis [10]. The identification of migraine biomarkers would help to increase the accuracy in its diagnosis, to improve the knowledge of the underlying pathogenic mechanisms, and to predict its progression and response of a therapeutic intervention [11].

Transcranial ultrasound (TCS) is a noninvasive imaging technique, harmless and easy-to-perform, with a demonstrated utility for the visualization of deep brain structures, such as the substantia nigra (SN), the raphe nuclei or the third ventricle. In particular, TCS has proven its usefulness in the diagnostic workout of movement disorders, such as Parkinson's Disease (PD), where approximately 90% of patients present with hyperechogenicity of the substantia nigra, now demonstrated biomarker for the prodromal phase of Parkinson’s disease (PD) [12, 13].

The aim of the current study was to assess the usefulness of TCS for migraine diagnosis. We hypothesize that the echogenicity of the PAG and RN is increased in patients with migraine, as a marker of structural damage, and consequent migraine chronification.

## Material and methods

### Study design and patient selection

This is a cross-sectional study conducted at the Headache Unit of the Hospital Universitari Germans Trias i Pujol, from March 2020 to June 2022. A prospective recruitment was carried out among those patients evaluated in the unit with a diagnosis of migraine according to the ICHD-3. Patients with a known neurological disease other than migraine were excluded from the study. The coexistence of other primary headaches was not an exclusion criterion in the patients with migraine group.

For comparative purposes, a group of control subjects, without diagnosis of migraine or other neurological pathologies, and with comparable age and sex distribution to the cases, was also evaluated. Control subjects were prospectively recruited from non-blood relatives of patients included in the study, as well as from healthy volunteers. A detailed interview, conducted by a headache-expert neurologist (LD), was performed to ensure that controls had no history of migraine or other primary headaches. Subjects who were on antidepressant treatment or treatment for chronic pain were excluded.

This study was approved by the Research Ethics Committee of Hospital Germans Trias i Pujol (PI-20–081). All subjects signed the informed consent form for participation in the study and use of their clinical data for research purposes.

### Clinical variables

Demographic variables and comorbidities were collected in all the subjects included in the study. In migraine patients, disease duration, characteristics and frequency of migraine (headache days per month, migraine days per month), and symptomatic treatment were recorded. Disability and quality of life associated with migraine were assessed using the Migraine Disability Assessment Scale (MIDAS) [14], the Headache Impact Test (HIT-6) [15] and the version 2 of the Migraine-Specific Quality-of-Life Questionnaire (MSQV2.19) [16]. The Hospital Anxiety and Depression Scale (HADS) [17] was completed.

### Transcranial sonography

**Fig. 1:**
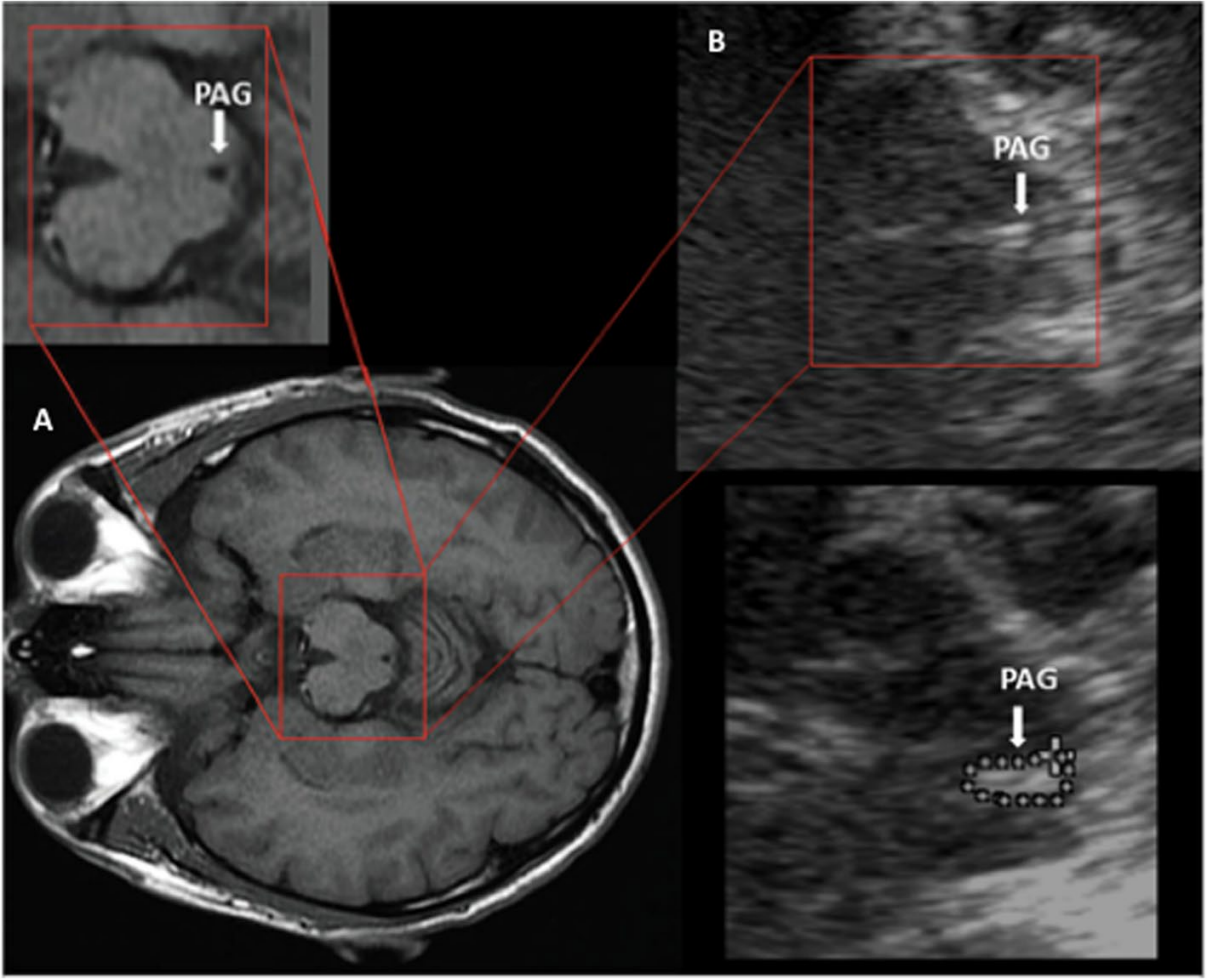
Magnetic resonance and transcranial sonography images of midbrain axial sections showing the periacueductal gray matter. The TCS images show images from our sonographic laboratory obtained with an ultrasound system for clinical application (Philips Affiniti 70 ultrasound machine). **A** MRI of the axial section at the level of the midbrain. The square box shows the area corresponding to the magnified TCS image shown in B. **B** TCS image of the axial midbrain section showing the area of the periaqueductal gray matter echogenicity

TCS was performed in all participants by a neurologist (DV) experienced in performing and interpreting transcranial sonography of deep brain structures [14, 15]. This neurologist was not related with the Headache Unit and was blinded to clinical data. A 2 MHz phased-array transducer was used (Philips Affiniti 70 ultrasound machine), with a penetration depth of 14 cm and a dynamic range of 45–55 db. Image brightness and time-gain compensation were predetermined and were not changed across the study. Tissue harmonic imaging settings were applied to increase the tissue contrast. The examination was performed from both sides of the head using the transtemporal bone window to evaluate the mesencephalic and the thalamic plane. Images were analyzed in two different ways. First, during the performance of TCS we made a bedside analysis. The PAG was defined, as observed by TCS, as a structure surrounding the aqueduct of Sylvius, with a higher echogenicity than the cerebrospinal fluid signal observed in the aqueduct. The PAG area was manually delimited and measured (Fig. 1). The echogenicity of the right and left RN was also evaluated. The RN is usually seen as a small white dot near the brainstem midline, posterior to the substantia nigra (SN), which is isoechogenic to the basal cisterns. When observed, the area of echogenicity of RN was manually delimited and measured. For internal validation purposes, other deep brain structures were also evaluated. Substantia nigra (SN) hyperechogenicity was considered if the area of SN echogenic signal area was equal to or greater than 0.20 cm2, following the cut-off values usually used with similar ultrasound systems [18]. The echogenicity of the brainstem raphe nuclei was graded as hypoechogenic when this midline structure in the midbrain was interrupted or not visible [19]. The width of the third ventricle (IIIv) was measured by taking the minimum transverse diameter in the thalamic plane and the right and left frontal horn of the lateral ventricles in the same plane. Secondly, the TCS images were de-identified and subsequently analyzed with the medical image viewer Horos by a second blinded explorer (SRG), previously trained. Horos is a free and open source code software (FOSS) program that is distributed free of charge under the LGPL license at Horosproject.org and sponsored by Nimble Co LLC d/b/a Purview in Annapolis, MD USA. All the structures previously evaluated in the bedside TCS were again assessed by means of Horos, with the same methodology. In addition, this program allows to quantify the intensity of the echogenicity of a region of interest (ROI). We manually outlined the area of echogenicity of the PAG and the RN, and the program gave us a histogram of each ROI, with the mean, the minimum and maximum echogenicity (unnamed units) of this region of interest.

### Statistical analysis

Results are described as median with interquartile range (IQR: 25^th^; 75^th^ percentiles) or with absolute frequencies and percentages, with inferential statistical analyses between groups by means Fisher’s exact test or Mann–Whitney U test for qualitative or quantitative variables respectively. Capacity of discrimination from PAG echogenicity between controls and patients was assessed by means of the area under the ROC curve (AUC) and its 95% confidence interval (95%CI). To analyze the morphologic changes within the PAG and the RN, we performed a texture analysis of both structures, comparing the extracted variables from histograms of their echogenicity, obtained from the Horos viewer, from patients with migraine and controls. The magnitude of the intensity of echogenicity was evaluated with the mean ROI, while the heterogeneity of this intensity was estimated with the coefficient of variation. Accordingly, not only the intensity of the echogenicity but also its texture could be evaluated jointly. Differences were considered statistically significant for a two-sided type I error of 0.05. SPSS Version 26 (IBM Corp. Armonk, NY. USA) was used for all statistical analyses.

## Results

We included 115 subjects (65 patients with migraine and 50 controls). Among patients with migraine, 39 (60%) had CM and 26 (40%) EM. Table 1 shows demographic and clinical characteristics of all participants. Neither statistical nor numerical differences were observed among groups regarding age and sex. Median disease duration was 29 (IQR: 19; 40) years for CM patients, with a median of 18 (IQR: 14; 27) days of migraine per month and 28 (IQR: 24; 30) days of headache per month. Regarding EM, median disease duration was 30 (IQR: 22; 40) years, with a median of 9 (IQR: 2; 12) days of migraine per month and 9.5 (IQR: 3; 12) days of headache per month.

**Table 1:** Demographic and clinical characteristics of patients with migraine and controls

	**Controls (*n* = 50)**	**Migraine patients (*n* = 65)**	**CM (*n* = 39)**	**EM (*n* = 26)**	***p* value (C vs M)**	***p* value (CM vs EM)**
Age (years)	43.50 [28.00;65.00]	45.00 [38.00;53.00]	45.00 [38.00;53.00]	45.00 [38.00;54.00]	0.954	0.981
Sex, female (*n*,%)	34 (68%)	47 (72.3%)	27 (69.23%)	20 (76.92%)	0.682	0.579
Disease duration (years)	-	30.00 [20.00;40.00]	29.00 [19.00;40.00]	30.00 [22.00;40.00]	-	0.384
Duration of CM (months)	-	-	60.00 [40.00;120.00]		-	NA
Days of migraine per month	-	14.00 [10.00;19.00]	18.00 [14.00;27.00]	9.00 [2.00;12.00]	-	< 0.001
Days of headache per month	-	20.00 [12.00;30.00]	28.00 [24.00;30.00]	9.50 [3.00;12.00]	-	< 0.001
NSAIDs overuse (n,%)	-	-	11 (28.21%)	1 (3.85%)	-	0.02
Triptans overuse (n,%)	-	-	12 (30.77%)	7 (26.92%)	-	0.787
MIDAS, total score	-	60.00 [20.00;96.00]	84.00 [56.00;122.00]	21.00 [3.50;36.50]	-	< 0.001
HIT-6		67.00 [62.00;70.00]	68.00 [65.00;70.00]	64.00 [55.50;67.00]	-	0.004
MSQV2.19		42.85 [30.00;65.58]	34.29 [22.85;51.43]	63.58 [46.43;88.58]	-	< 0001
HADS Anxiety, score	-	9.00 [6.00;14.00]	11.00 [7.00;16.00]	7.50 [4.00;10.00]	-	0.002
HADS Depression, score		6.00 [4.00;10.00]	9.00 [5.00;12.00]	4.00 [3.00;8.00]	-	0.001

*C* Controls, *M* Migraine patients, *CM* Chronic migraine, *EM* Episodic migraine, *NSAIDs* Nonsteroidal anti-inflammatory drugs, *MIDAS* Migraine Disability Assessment Scale, *HIT-6* Headache Impact Test, *MSQV2.19* Version 2 of the Migraine-Specific Quality-of-Life Questionnaire, *HADS* Hospital Anxiety and Depression Scale

### Transcranial sonography findings

Forty-eight (96%) controls and 64 (98.5%) patients with migraine had an adequate transtemporal bone window to assess the echogenicity of the PAG. The echogenicity of the right RN was assessed in 65 (56,52%) of participants and echogenicity of the left RN in 63 (54,78%) because of an inadequate bone window.

According to the bedside assessment, the median area of the PAG was higher in patients with CM compared to EM and controls, with 0.15 (IQR: 0.12;0.22), 0.11 (IQR: 0.10;0.14) and 0.12 (IQR: 0.09;0.15) cm^2^, respectively (*p* = 0.043). Also, we found similar results when performing the analysis with the Horos medical viewer (Table 2).

**Table 2:** Sonographic variables of patients with migraine and controls

	**Controls (*n* = 50)**	**Migraine patients (*n* = 65)**	**CM (*n* = 39)**	**EM (*n* = 26)**	**Controls vs migraine patients**	**Controls vs CM**	**Controls vs EM**	**CM vs EM**
**Bed-side analysis**								
PAG area	0.12 [0.09;0.15]	0.13 [0.11;0.17]	0.15 [0.12;0.22]	0.11 [0.10;0.14]	0,043*	0,004*	0,895	0.017
Right RN	0.06 [0.05;0.08]	0.05 [0.04;0.07]	0.05 [0.04;0.07]	0.06 [0.05;0.07]	0,281	0,336	0,369	0.940
Left RN	0.07 [0.05;0.09]	0.07 [0.05;0.08]	0.07 [0.05;0.09]	0.06 [0.05;0.08]	0,868	0,962	0,782	0.763
Right SN area	0.09 [0.07;0.12]	0.10 [0.08;0.13]	0.11 [0.08;0.14]	0.10 [0.07;0.12]	0,082	0,013*	0,935	0.127
Left SN area	0.11 [0.08;0.13]	0.11 [0.09;0.13]	0.11 [0.09;0.14]	0.10 [0.08;0.13]	0,793	0,478	0,697	0.308
IIIv size	0.33 [0.27;0.45]	0.29 [0.21;0.44]	0.29 [0.21;0.46]	0.30 [0.25;0.38]	0,092	0,13	0,205	0.914
Right horn LV	1.40 [1.26;1.55]	1.45 [1.32;1.60]	1.54 [1.40;1.60]	1.39 [1.25;1.47]	0,367	0,06	0,535	0.026*
Left horn LV	1.50 [1.32;1.68]	1.49 [1.35;1.67]	1.49 [1.31;1.60]	1.54 [1.35;1.68]	0,771	0,868	0,462	0.352
**Horos analysis**								
PAG area	0.13 [0.12;0.18]	0.16 [0.13;0.20]	0.16 [0.13;0.20]	0.17 [0.12;0.20]	0,038*	0,04*	0,191	0.650
PAG echogenicity (intensity)	109.56 [83.30;122.64]	93.84 [71.34;117.26]	90.57 [70.87;117.26]	101.70 [76.44;115.17]	0,07	0,035*	0,457	0.221
PAG echogenicity (CV)	25.40 [20.08;31.84]	29.26 [23.69;35.02]	32.24 [25.74;35.83]	25.84 [21.78;30.95]	0,12	0,009*	0,688	0.003*
Right SN area	0.10 [0.08;0.14]	0.12 [0.10;0.15]	0.13 [0.09;0.14]	0.12 [0.10;0.15]	0,006*	0,09*	0,003*	0.260
Rigth SN echogenicity (intensity)	82.87 [68.57;95.34]	79.76 [63.52;100.93]	79.76 [59.48;99.03]	80.80 [67.74;101.18]	0,96	0,769	0,8	0.743
Rigth SN echogenicity (CV)	27.08 [20.92;33.90]	28.13 [22.45;37.52]	29.27 [20.37;40.22]	27.48 [23.30;34.00]	0,545	0,624	0,608	0,940
Right RN area	0.07 [0.07;0.08]	0.08 [0.07;0.09]	0.08 [0.07;0.09]	0.08 [0.07;0.10]	0,954	0,96	0,838	0.612
Rigth RN echogenicity (intensity)	85.54 [67.46;104.89]	69.61 [53.52;97.05]	75.59 [53.52;86.33]	61.56 [56.12;100.41]	0,062	0,077	0,15	1.000
Rigth RN echogenicity (CV)	23.85 [20.04;29.51]	28,79 [20.59;31.83]	28.92 [22.10;32.81]	28.30 [26.43;30.69]	0,135	0,193	0,193	0.780
Left SN area	0.10 [0.08;0.12]	0.11 [0.09;0.16]	0.11 [0.09;0.16]	0.12 [0.09;0.16]	0,01*	0,027*	0,033*	0.632
Left SN echogenicity (intensity)	82.17 [55.90;102.12]	78.06 [62.68;95.60]	74.35 [64.13;94.45]	80.49 [61.23;106.43]	0,972	0,827	0,709	0.500
Left SN echogenicity (CV)	26.39 [20.16;29.87]	28.43 [21.77;35.20]	28.43 [23.07;32.66]	27.07 [21.41;36.30]	0,163	0,179	0,348	0.848
Left RN area	0.08 [0.05;0.09]	0.07 [0.05;0.10]	0.07 [0.06;0.08]	0.08 [0.05;0.11]	0,796	0,837	0,377	0.675
Left RN echogenicity (intensity)	89.34 [73.36;108.69]	8.,45 [59.84;96.68]	85.47 [68.49;96.60]	76.00 [58.66;106.75]	0,235	0,268	0,377	0.884
Left RN echogenicity (CV)	25.18 [22.07;37.25]	29.86 [22.80;55.92]	35.51 [24.82;64.38]	23.97 [21.06;31.34]	0,383	0,124	0,679	0.051

*CM* Chronic migraine, *EM* Episodic migraine, *SN* Substantia igra, *PAG* Periaqueductal gray matter, *IIIv* Third ventricle size, *LV* Lateral ventricle, *CV* Coefficient of variation, *RN* Red nucleus.*p value < 0.05

PAG intensity of echogenicity was significantly lower in CM patients than in controls, 90.57 (IQR: 70.87;117.26) vs 109.56 (IQR: 83.30;122.64), *p* = 0.035. EM patients and controls showed similar intensity of the PAG echogenicity (*p* = 0.457).

In addition, CM patients had higher coefficient of variation of the PAG echogenicity (*p* = 0.009) compared to EM and controls (Table 2). The area of PAG echogenicity and the intensity of the echogenicity of the PAG were similar among CM or EM patients with and without NSAIDs or triptans overuse (Supplementary Table 1).

No differences were observed regarding the area or intensity of RN echogenicity among groups, neither in the bedside analysis or in the analysis with the Horos medical viewer (Table 2). The area of SN echogenicity was higher in migraine patients than in controls, being the patients with CM those with a higher SN echogenicity area (right SN: CM 0.13[0.09;0.14] cm2, EM 0.12[0.10;0.15] cm2, controls 0.12[0.10;0.15]cm2;*p* = 0.006; left SN: CM 0.11[0.09;0.16], EM 0.12[0.09;0.16], controls 0.11[0.09;0.16]; *p* = 0.01) (see Table 2).

The AUC in the ROC analysis to classify control subjects and patients with migraine for the area of the PAG and for the intensity of the PAG echogenicity was 0.61 (95%CI 0.51; 0.72) and 0.60 (95%CI 0.50; 0.71), respectively; and the AUC for control subjects and CM for the coefficient of variation of the intensity of the PAG echogenicity was 0.59 (95%CI 0.48; 0.70).

## Discussion

The main findings of the current study are that patients with CM presented structural changes of the PAG with a larger area, a lower intensity and a higher heterogenicity of its echogenicity, compared to patients with EM and controls.

Our results suggest that the structure of the PAG is altered in patients with CM, compared to controls and, interestingly, to EM patients. Also, EM patients and controls are quite similar in terms of PAG and RN echogenicity. To our knowledge, this is the first case–control study that evaluates the PAG and the RN by means of TCS. If our results are replicated in further studies, the echogenicity of the PAG might emerge as a biomarker of migraine chronification.

Although the pathophysiology of migraine is still under investigation, the PAG is an important structure that modulates nociceptive transmission. It is thought that the function of brainstem pain modulating circuits contributes to the maintenance of chronic neuropathic pain, even predisposing individuals to develop chronic pain [6]. PAG is activated during migraine attacks, and repeated migraine attacks could lead to free radical damage associated with hyperemia and consequent iron deposition in this area [9]. Almost 30% of the CM patients included in our study presented an overuse of anti-inflammatory drugs, which is an important factor for the chronification process. Although, in our cohort, CM patients with and without medication overuse showed a similar PAG area and PAG intensity, further studies should consider this issue. MRI studies in migraine patients showed the presence of iron deposits in the PAG and RN. The size of these deposits correlates with the frequency of migraine attacks, their intensity, and the time course of the disorder [8, 9]. It has therefore been hypothesized that iron deposition may reflect progressive dysfunction of the PAG and other brainstem structures related to normal antinociceptive function, contributing to migraine chronification [20]. In a recent study, functional biomarkers, including PAG networks, seems to be the most important MRI features in classifying migraine patients from controls [21].

From a sonographic point of view, CM and EM patients seems to behave different in our study, suggesting that the pathophysiology of both entities is different. We did not find a cutoff point of the PAG with enough sensitivity and specificity to use it as an aid in the diagnosis migraine patients. Further studies with larger sample to improve the representativeness of migraine patients might evaluate this aspect.

We found no differences among patients with migraine and controls regarding the RN echogenicity. This could be explained, at least partly, because we only observed this brainstem structure in around 55% of participants. It could be also possible that the RN has a lower implication in the pathophysiology of migraine. Functional MRI studies as well as prospective studies with TCS in patients with migraine could help to clarify this issue.

TCS, a noninvasive imaging technique with a demonstrated utility for the visualization of deep brain structures, has proven its usefulness in the assessment of movement disorders, such as PD, where approximately 90% of patients present with hyperechogenicity of the SN [17]. TCS has been rarely used in patients with migraine. We previously reported that the hypoechogenicty of the raphe nuclei is more prevalent in patients with migraine than in controls [22], concordant with results from previous studies [23–26] and supporting the role of the raphe nuclei in migraine. In the current study, the first that assess the PAG by means of TCS, we also support the role of the PAG in the pathophysiology of migraine.

The significance of the sonographic changes that we observed in the PAG in patients with migraine needs to be address. The well-known hyperechogenicity of the SN observed in PD, seems to be due to different structural changes. Different imaging investigations, experimental studies in animal models and postmortem analysis in humans, may support the hypothesis that alterations in local iron deposition and changes in cellular composition in the SN lead to its hyperechogenicity [27]. In general, the echogenicity of any structure depends on its acoustic impedance and on the relationship with adjacent structures. An increase in the echogenicity of deep brain structures may result from the composition of the components of neurons, glia, and connecting fibers. On the other hand, the accumulation of heavy metals, such as iron, copper or manganese, can result in an increase in echogenicity. The hyperechogenicity of the SN in PD has been associated with an increase in the content of heavy metals such as iron, copper or manganese [28] and oxidative damage [29]. Contrary of what we suspected, intensity of echogenicity in PAG was lower in CM patients than controls and EM. This could reflect that, other mechanisms, different to iron deposition, are also present, leading to a different composition in the tissue of this particular area implicated in pain modulation. Further studies are needed to address this point.

Unexpectedly, we also found that the area of SN echogenicity was higher in migraine patients than in controls, especially in CM patients. This finding was not previously reported and further studies are needed to confirm our results.

Some limitations of our study include the relatively small sample size, the absence of comparison of the echogenicity of the PAG with previously reported imaging biomarkers such as the MRI iron deposits and, finally, the fact that the TCS is an explorer-dependent technique.

New tools are needed to reduce the delay in the diagnosis of migraine, to facilitate the initiation of treatment at its earlier stages and to detect the predictors of response to treatments. Ideally, these tools should be accessible to the network of physicians involved in the diagnosis of migraine, cheap and non-invasive. In this direction, if other groups replicate our findings, TCS might be a useful tool in the diagnosis of migraine. Longitudinal studies in CM patients could also help to elucidate the role of the TCS in the prediction of response to new treatments, such as anti-calcitonin gene-related peptide monoclonal antibodies, and others recently approved in migraine.

## Conclusions

The echogenicity of the PAG, evaluated by means of TCS, seems to be a marker of disease chronification in migraine. If confirmed by other groups, our findings suggest that TCS, a cheap, quick and easy-to-apply technique, could be useful for the diagnosis and prognosis of patients with migraine.

## Supplementary Information

**Additional file 1: Supplementary Table 1.**

## Data Availability

The datasets used and analysed during the current study are available from the corresponding author on reasonable request.

## Abbreviations

PAG: Periaqueductal gray matter
RN: Red nucleus
TCS: Transcranial ultrasound
ICHD-3: International Classification of Headache Disorders
CM: Chronic migraine
EM: Episodic migraine
MIDAS: Migraine Disability Assessment Scale
HIT-6: Headache Impact Test
MSQV2.19: Migraine-Specific Quality-of-Life Questionnaire HADS: Hospital Anxiety and Depression Scale
SN: Substantia nigra
IIIv: Third ventricle
AUC: Area under curve
IQR: Interquartilic range
ROI: Region of interest
