# Supplementary material for: Periaqueductal gray matter echogenicity as a marker of migraine chronification: a case control study
Source: J Headache Pain. 2023 Apr 17;24(1):41. doi: 10.1186/s10194-023-01576-3 (PMC10108492; doi:10.1186/s10194-023-01576-3)
Supplement: Supplementary file 1 — Additional file 1: Supplementary Table 1. [file 10194_2023_1576_MOESM1_ESM.docx]

**Supplementary Table 1. Sonographic findings in CM and EM patients with and without medication overuse.**

| **CM patients (n=39)** | | | | | | |
| --- | --- | --- | --- | --- | --- | --- |
|  | **NSAIDs overuse** | | p value | **Triptans overuse** | | p value |
|  | **No**  **(n=28)** | **Yes**  **(n=11)** |  | **No**  **(n=27)** | **Yes**  **(n=12)** |  |
| **PAG area** | 0.15  (0.12;0.22) | 0.15  (0.12;0.17) | 0.642 | 0.13  (0.11-0.21) | 0.15  (0.12-0.22) | 0.485 |
| **PAG intensity** | 93.84  (71.23;121.05) | 83.83  (66.65;92.55) | 0.975 | 111.51  (79.32-144.65) | 84.41  (66.75-98.94) | 0.106 |
| **EM patients (n=26)** | | | | | | |
|  | **No**  **(n=25)** | **Yes**  **(n=1)** |  | **No**  **(n=19)** | **Yes**  **(n=7)** |  |
| **PAG area** | 0.11  (0.10-0.14) | 0.16  (0.16-0.16) | 0.40 | 0.11  (0.09-0.14) | 0.16  (0.10-0.22) | 0.27 |
| **PAG intensity** | 101.88  (76.35-124.51) | 101.70  (101.70-101.70) | 1.00 | 102.87  (76.44-133.84) | 101.61  (71.34-112.84) | 0.57 |

CM= chronic migraine, EM= episodic migraine; NSAIDs= nonsteroidal anti-inflammatory drugs; PAG= periaqueductal gray matter.
